# General practitioners’ views on managing knee osteoarthritis: a thematic analysis of factors influencing clinical practice guideline implementation in primary care

**DOI:** 10.1186/s41927-018-0037-4

**Published:** 2018-10-26

**Authors:** Thorlene Egerton, Rachel K Nelligan, Jenny Setchell, Lou Atkins, Kim L Bennell

**Affiliations:** 1 Centre for Health, Exercise and Sports Medicine, The University of Melbourne, Melbourne, Australia; 2 School of Health and Rehabilitation Sciences, The University of Queensland, Brisbane, Australia; 3 Centre for Behaviour Change, University College London, London, UK

**Keywords:** Knee osteoarthritis, Primary care, Clinical guidelines, General practitioner, Qualitative

## Abstract

**Background:**

Osteoarthritis (OA) is diagnosed and managed primarily by general practitioners (GPs). OA guidelines recommend using clinical criteria, without x-ray, for diagnosis, and advising strengthening exercise, aerobic activity and, if appropriate, weight loss as first-line treatments. These recommendations are often not implemented by GPs. To facilitate GP uptake of guidelines, greater understanding of GP practice behaviour is required. This qualitative study identified key factors influencing implementation of these recommendations in the primary-care setting.

**Methods:**

Semi-structured interviews with eleven GPs were conducted, transcribed verbatim, coded by two independent researchers and analysed with an interpretive thematic approach using the COM-B model (Capability/Opportunity/Motivation-Behaviour) as a framework.

**Results:**

Eleven themes were identified. Psychological capability themes: knowledge gaps, confidence to effectively manage OA, and skills to facilitate lifestyle change. Physical opportunity themes: system-related factors including time limitations, and patient resources. Social opportunity theme: influences from patients. Reflective motivation themes: GP’s perceived role, and assumptions about people with knee OA. Automatic motivation themes: optimism, habit, and unease discussing weight. The findings demonstrated diverse and interacting influences on GPs’ practice.

**Conclusion:**

The identified themes provide insight into potential interventions to improve OA management in primary-care settings. Key suggestions include: improvements to OA clinical guidelines; targeting GP education to focus on identified knowledge gaps, confidence, and communication skills; development and implementation of new models of service delivery; and utilising positive social influences to facilitate best-practice behaviours. Complex, multimodal interventions that address multiple factors (both barriers and facilitators) are likely to be necessary.

## Background

Osteoarthritis (OA) is a highly prevalent, disabling condition ranked the eleventh highest contributor to global disability [1, 2]. Knee OA is mostly diagnosed and managed in family practice settings [3] and principally by general practitioners (GPs, i.e. family doctors) [4]. Clinical practice guidelines (CPGs) recommend diagnosing OA using clinical criteria without imaging, and facilitating self-management through education and the provision of advice on weight management and increasing physical activity [5]. However, care inconsistent with these recommendations has been identified in several countries and health care settings [6–11]. GPs tend to over-rely on imaging for diagnosing OA [12, 13], under-emphasise exercise and weight loss options in favour of drug and surgical management [6, 9, 14], frequently refer for ineffective arthroscopic surgery [15], or refer for joint replacement surgery before an adequate trial of recommended conservative treatments [10, 14].

There is a need to develop effective strategies that facilitate GPs’ uptake of recommended OA management practice. Detailed behavioural analysis of the reasons behind the inadequate uptake will help inform implementation interventions [16]. There have been previous qualitative studies asking primary care practitioners about topics related to the provision of care for people with knee osteoarthritis [7, 17, 18] and osteoarthritis more generally [19–21]. Common findings include trivialising or normalising the problem, lack of knowledge/skills, and resource issues [22]. However, previous studies on the topic have not focussed in any depth on the barriers and facilitators to implementation of the priority recommendations currently identified as being the most underutilised in care globally [22]. The aim of this study was therefore to identify barriers and facilitators influencing whether GPs perform the activities of: 1) making a clinical diagnosis without imaging, 2) engaging patients in exercise and physical activity, and 3) engaging patients in weight loss. The study involved the systematic and comprehensive identification of behavioural drivers related to providing this care for people with knee osteoarthritis with the hope of uncovering new and useful additional findings. We used a novel framework to guide our classification and labelling of themes, and included a discussion of the results in the context of previous findings.

## Methods

This study is part of a larger project (PARTNER) to increase delivery of recommended knee OA management within Australian primary health care. All GPs provided informed consent to be interviewed and recorded. The reporting of this study adheres to the COnsolidated criteria for REporting Qualitative studies (COREQ) 32-item checklist [23].

### Design and theoretical framework

Semi-structured telephone interviews were used for data collection. An interpretive thematic analysis methodology was adopted with reference to the COM-B (Capability/Opportunity/Motivation-Behaviour) model [24] as a comprehensive framework for theme development. The COM-B model explains behaviour as resulting from interactions between physical and psychological capabilities, social and environmental opportunities, and motivators that can be either reflective (deliberate, conscious thought processes) or automatic (emotional or reactive). COM-B component definitions are provided in Table 4. The COM-B model has been used extensively in the design of behavioural interventions in a range of settings [25–27]. The COREQ-checklist was used to ensure transparent reporting of this study [23].

### Participants

A purposive sample of eleven GPs ensuring a range of practice sizes, age, metropolitan/regional locations and years of practice was recruited. Initially, GPs from The Victorian Primary Care Research Network database were provided with information on study aims and invited to volunteer. Snowballing was later used to identify additional participants to approach. During recruitment, the investigators iteratively monitored participant characteristics to ensure sufficient diversity for the purposive sampling. All eligible GPs (*n* = 11) who expressed interest in volunteering were included. GPs were eligible if they were practicing in a primary care setting and saw at least one patient with knee OA per month. The sample size was determined by the concept of theoretical saturation when iterative review of the data showed sufficient repetition and depth of COM-B and inductive themes [28].

### Procedure

The semi-structured interview guide, developed in collaboration with a behaviour change expert experienced in applying the COM-B model (LA) and a qualitative research expert (JS), incorporated all components of COM-B model (Table 1) and allowed further exploration of topics raised by participants.

**Table 1:** Semi-structured interview guide

Key activity	Questions and potential probes
GP makes, communicates and documents a diagnosis of osteoarthritis clinically (without imaging)	*How do you currently arrive at a diagnosis of knee OA?* − *Are you aware that guidelines recommend making a diagnosis clinically and without imaging?* − *How do you feel about making a clinical diagnosis of knee OA (without imaging)?* − *What would you or GPs in general need to know more about in order to be comfortable with making a clinical diagnosis of knee OA?* − *What would help encourage or support GPs in making a clinical diagnosis of knee OA?* *How do you think receiving a diagnosis of knee OA impacts on patients?* *Are there any issues around patient expectations that influence how you diagnose knee OA and how you communicate the diagnosis with patients?* *Do you currently document diagnosis of “knee OA” in patients’ records?*
GP provides education/advice to patients about the importance of general physical activity and regular strengthening and/or aerobic exercise during the consultation which is reinforced at later opportunities.	*What physical activity or exercise advice do you currently give to patients with knee OA?* *How confident do you feel when giving this advice?* *How important do you think it is to talk to your knee OA patients about physical activity and strengthening exercises?* *How do you think this information impacts on patients?* *Do you think GPs are familiar with the latest recommendations for physical activity in general and for exercise specifically for people with knee OA?* *Are there any additional skills or training that you would like to have regarding physical activity or exercise advice?* *Are there any other things that make it difficult for GPs to give this advice?* *Can you suggest any measures that would assist or support GPs in discussing general physical activity and targeted exercises with their knee OA patients?*
GP provides education/advice to patients either about the importance of maintaining a healthy weight or weight loss in the initial consultation which is reinforced at later opportunities (includes BMI measurement)	*What weight management advice do you currently give to patients with knee OA?* *How important do you think weight loss is for knee OA symptoms?* *How confident do you feel when giving weight loss advice?* *How do you think this information impacts on patients?* *Do you think GPs feel motivated to talk to patients about weight management / weight loss? What would increase or decrease their motivation?* *Are there any other things that make it difficult for GPs to give effective weight loss advice?* *Can you suggest any measures that would assist or support GPs in talking to patients about weight management / weight loss?* *Do you currently assess BMI with your knee OA patients?* − *How important is it that GPs assess BMI for knee OA patients?* − *What, if any, are the benefits to patients?* − *If you think GPs do not currently routinely assess BMI, what are the reasons for this? What shift of thinking is required?* − *What help or support would make it easier for GPs to assess BMI for their knee OA patients?* − *Are there any issues around patient expectation that influence whether BMI is assessed?*

A physiotherapist trained in qualitative interviewing (RN), conducted all interviews. Interviews were audio recorded, transcribed by an external company and checked for accuracy by RN. Field notes were taken. Digital transcripts were de-identified and stored securely. GPs were offered a $50 voucher for their participation. Interviewed GPs did not review their finalised transcripts.

Data were analysed by TE and RN and overseen by JS and LA. The systematic iterative approach used is detailed in Table 2. In summary, TE and RN both independently read all transcripts and generated codes, themes were inductively generated and revised, and these were then organised according to the COM-B model components. Data collection and analysis occurred concurrently.

**Table 2:** Thematic analysis stages

Stage	Description
I.	Initial familiarisation with the data – by RN and TE who listened to all audio files and read transcripts as they became available.
II.	Inductive coding of the data - RN and TE independently coded the data to identify recurrent patterns, common beliefs, barriers and enablers.
III.	Codes were discussed, and consensus reached - discussion between RN and TE, agreement reached on themes by grouping segments of code into broader categories (themes). Microsoft Excel spreadsheets used to help manage the data. In the instance of differing opinions input from JS (qualitative expert) was sought.
IV.	Themes refined and anchored to COM-B model framework – RN and TE jointly revised themes into overarching themes with codes within themes, and anchored these to the COM-B components through several iterations.
V.	Themes and codes reviewed, revised and agreed upon by all members of the research team and results summarised.

## Results

Table 3 outlines participant characteristics. Interviews ranged from 30 to 54 min. Analysis identified eleven themes (Table 4) from five of the six COM-B components. No themes were identified in the physical capability component.

**Table 3:** Demographic characteristics of participating general practitioners

GP	Sex	Years in practice	Metropolitan / regional	Size of practice (number of GPs)	Approximate number of knee OA patients per month
GP1	F	32	Regional	4	6
GP2	F	26	Metropolitan	6	10–20
GP3	F	22	Metropolitan	13	2
GP4	M	44	Regional	1	40
GP5	F	5	Regional	15	20
GP6	M	31	Regional	4	6
GP7	M	30	Regional	4	4
GP8	F	26	Metropolitan	3	30
GP9	F	6	Metropolitan	24	3 to 4
GP10	F	10	Metropolitan	5	1
GP11	M	6	Metropolitan	4	3 to 10

**Table 4:** Themes within the COM-B model

COM-B component	Theme	Code
*Definition*		
Psychological Capability	Knowledge gaps	Knowledge of OA disease processes and progression
*Knowledge or psychological skills to engage in the necessary mental processes*		
		Adequate knowledge about making diagnosis without imaging
		Knowledge of effective exercise and weight loss treatments
	Skills to facilitate lifestyle change	Communication skills
		Facilitation of behaviour change
	Confidence to effectively manage OA	Making the diagnosis without x-ray
		Delivering lifestyle interventions
Physical Opportunity	System-related factors	Time availability
*Opportunity afforded by the environment*		Access to other services for exercise and weight management advice (including cost and ease of referral)
		Clinic software
		Lifestyle treatments recommended for all chronic disease patients
	Patient resources	Ease of access
Social Opportunity	Influences from patients demands and expectations	
*Opportunity afforded by interpersonal influences, social and cultural norms that impact the way we think about things*		
Reflective Motivation	GP’s perceived role	Paternalistic role
*Reflective processes involving self-conscious intentions, beliefs regarding good and bad, self-talk*		
		Use patient-centred approaches
	Assumptions about people with knee OA	Diagnosis of OA may foster fear avoidance behaviours
		Patient motivation to adopt lifestyle change
Automatic Motivation	Optimism	Effectiveness of non-drug conservative treatment options
*Automatic processes involving emotion, desires, impulses*		
	Habit	
	Unease discussing weight	

### Psychological capability

Three themes were identified within the COM-B component ‘psychological capability’. These were ‘knowledge gaps’, ‘confidence to effectively manage OA’, and ‘skills to facilitate lifestyle change’.

The first theme ‘knowledge gaps’ was based on GP comments relating to their knowledge about disease processes, diagnosis and best practice. In contrast to contemporary understandings [29], most GPs said they described OA to their patients as simply a problem of cartilage degeneration, joint space narrowing (on x-ray) or *“wear and tear” [GP11]* and frequently expressed beliefs that symptoms will progress, and that surgery is inevitable:

*“They know it’s permanent and…we’re looking at knee replacements in the future” [GP10].*

While most GPs demonstrated an understanding that x-ray findings do not typically match clinical presentation, and some were aware that imaging is not needed to reach a diagnosis of knee OA, some had a knowledge gap in this area. Some GPs reported referring for x-ray whenever knee OA is suspected, for example: *“(I) wouldn’t make a diagnosis without confirmatory imaging” [GP4]*. The same GP stated a belief imaging was required for diagnosis: “*Not without imaging…there can be other causes of the knee pain…” [GP4]*. Despite their statements to the contrary, most of the interviewed GPs also stated a preference to use imaging to “confirm” diagnosis, and some said that imaging helps clarify disease severity.

Several comments indicated that GP knowledge of exercise and weight-loss treatments is sometimes inaccurate or inadequate. For example, in contrast to current guidelines, some GPs thought land-based exercises and joint-loading activities are detrimental, that exercise in water is the only option they can recommend, and people with knee OA considered overweight may be unable to exercise at all:

*“There’s a really difficult group…they can’t exercise…they’re so overweight…unless they’re prepared to go to a pool there’s often many barriers for them.” [GP3].*

A few GPs were dubious about the effect of exercise and weight-management advice on reducing symptoms:

*“I haven’t found that it [exercise] is particularly helpful. I haven’t had anyone coming and raving to me saying ‘Oh I felt brilliant after a swim – my knee feels amazing’. It never happens.” [GP9].*

The second psychological capability theme identified was ‘confidence to effectively manage OA’. A few GPs demonstrated reduced *confidence* with making a diagnosis without imaging, despite having the *knowledge* that x-ray findings are not needed*.* For example, they said they relied on x-ray investigations for knee OA diagnosis due to low trust in their own diagnostic abilities and to “*confirm*” diagnosis: *“I have to admit that I’m not that confident” [GP9].* Reduced confidence with providing suitable exercise and weight loss advice was also demonstrated with some reporting it as their reason for referring to allied health professionals. Most recognised a need for tailored GP education to improve their confidence:

*“A physio showing us a few exercises…that would be a very good thing… for us to feel a bit more confident.” [GP1].*

The final psychological capability theme was about having ‘skills to facilitate lifestyle change’. All GPs reflected on the importance of having highly effective communication skills. For example:

*“Most of a GP’s life is about understanding the patient’s difficulties and barriers and motivations and then acting out your advice in a way that, hopefully, helps that patient” [GP6].*

The interviewed GPs acknowledged challenges of facilitating behaviour change and most felt they lacked skill in promoting readiness and motivation for these lifestyle treatments:

*“The problem is how do you actually get people to do this stuff…how do you tell them what the right thing to do is?” [GP3].*

### Physical opportunity

Two physical opportunity themes were identified: ‘system-related factors’ and ‘patient resources’. Regarding ‘system-related factors’, time pressure was discussed as a major barrier. Most GPs said they felt unable to individualise weight management and develop exercise plans within the appointment time. For example:

*“The bigger issue is, I feel I don’t have enough time to really give it in a way that I’m completely satisfied with” [GP5].*

All interviewed GPs said that OA was often only one part of a patient’s complex multi-morbidity and having time to devote to discussing OA management feels like a *“luxury” [GP3]*. However, most GPs also acknowledged that lifestyle treatments benefited other chronic conditions, which they said was a facilitator to finding the time for such treatments. One GP stressed the importance of longer consultations:

*“I’m a very passionate believer that long consults are of great benefit to taking a comprehensive history…and making a confident diagnosis” [GP8]*.

Another system-related factor identified by the interviewed GPs was limited access to other services and their associated costs. All participants expressed concerns regarding financial cost to patients when considering referral to other services:

*“There may be costs for patients to engage in these programs and obviously that can be a barrier” [GP11]*.

Others stated barriers to utilisation of support services such as community-based rehabilitation programs included lack of availability in remote locations and long waiting lists. Most of the GPs saw government-subsidised allied health visits as a system-related facilitator to utilisation of services that support exercise and weight loss. For example:

*“It’s only through a chronic disease management plan that a patient can get funded allied health” [GP3].*

Most participating GP’s identified changes to clinical practice information technology as potential system-related facilitators, particularly to diagnosing knee OA without imaging. Suggestions offered included building specific prompts into clinic software. In contrast, one GP was sceptical about the benefit of such tools:

*“The reality is those tools [checklists] exist for lots of conditions and we never use them, you know, because they’re not really practical” [GP3].*

‘Patient resources’ was the other physical opportunity theme. Having access to customisable, printable patient resources was suggested as a facilitator to GP-patient communication about both diagnosis and management options. Interestingly, one GP thought that suitable patient resources are already available (e.g. Arthritis Australia resources) commenting the issue is not a lack of resources but awareness of them:

*“The resources that are already out there, are they actually being used appropriately... I’m not convinced” [GP5].*

Some suggested that having patient resources embedded within current practice software or routines would increase their use by GPs.

### Social opportunity

One social opportunity theme was identified: ‘influences from patients demands and expectations’. Interviewed GPs expressed concern that poor patient health literacy in chronic disease management and patients’ beliefs about knee OA treatment efficacy negatively influenced how exercise and weight management were discussed. Some mentioned patients often have their own ideas on management, gained from media sources, family or friends. This could understandably be problematic if they primarily involve passive treatments such as supplements and injections. Shifting patients’ mind-sets to active participation in management and making lifestyle changes was reported as challenging and time consuming for GPs:

*“They often come in not wanting to talk about exercise and losing weight. They want to come in and say what they’ve read in a recent article” [GP3].*

In addition, interviewed GPs reported patient “*expectation*” *[GP11]* and “*pressure*” *[GP6]* had substantial influence on their decision to order x-ray investigations. One GP reflected:

*“I do agree with the guideline...but it is hard when patients demand” [GP9]*.

### Reflective motivation

Two themes were identified: ‘GPs’ perceived role’ and ‘assumptions about people with knee OA’. Those interviewed had varied beliefs about the GP role in OA management. Different beliefs appeared to influence the level of engagement in providing exercise and weight management advice. Some GPs demonstrated a paternalistic approach to care, seeing their role as diagnosing and giving specific treatment advice:

*“I take a history…I might do further investigations. If the history and the physical examination fitted, [I] tell the patients this is what they have…” [GP3].*

A few GPs said they managed OA with a patient-centred approach, discussing the benefit of working with patients to make decisions about lifestyle change:

*“We certainly want to play our role and help improve [their] symptoms but [they] are actually going to be the most important person in terms of determining what happens from here” [GP5].*

The second theme was ‘assumptions about people with knee OA’. Interviewed GPs stated concerns with giving patients a knee OA diagnosis because they *assumed* patients would have negative connotations associated with the label:

*“It’s a difficult diagnosis to receive. Patients have a fear of being diagnosed. It’s disappointing for them” [GP10]*.

One GP said a diagnosis can foster fear-avoidance behaviours, including reduced activity, as patients may believe that activity/exercise will cause further damage. Most GPs were pessimistic about their patients’ abilities to make lifestyle changes to address their knee OA, assuming patients are not capable of making the required changes. One GP said firmly:

*“There are a lot of patients who are lazy…won’t carry out instructions and the recommendation to exercise” [GP4].*

Another reflected:

*“Giving people information is important but how much of it do they take on board? I guess it just varies according to the motivation of the patient” [GP3].*

As a result of these assumptions, the interviewed GPs demonstrated reduced motivation to communicate the diagnosis and pursue exercise and weight-loss conversations with their knee OA patients.

### Automatic motivation

Three themes were identified from the GPs’ discussions: ‘optimism’, ‘habit’ and ‘unease discussing weight’. GP optimism about OA management was suggested to facilitate provision of exercise and weight loss advice. A few of the GPs interviewed said they believed knee OA is a condition that can be successfully managed. They discussed the importance of conveying to patients that the diagnosis is not all negative, and try to promote management options with optimism and *“hope” [GP3]*. They argued that delivering a relatively positive prognosis to patients facilitated uptake of lifestyle changes:

*“Acknowledging that it’s difficult but that even in the face of difficulty there are many things that can help slow the progression” [GP3].*

The influence of ‘habit’ was conveyed as a barrier. For example, the GPs discussed that referral for x-ray was the way things had always been done:

*“It’s usually a two-step process: the patient comes in and you get the history, then they come back to discuss the investigations” [GP3]*.

A sense of ‘unease discussing weight’ with patients was conveyed by all but one GP. GPs interviewed acknowledged that weight loss (when someone is overweight) is important but felt that it was a sensitive topic. Most said they were afraid of upsetting their patients and this resulted in a temptation to avoid the discussion:

*“It’s very demoralising for some patients. It creates an avoidance. I’m sure that’s why we don’t raise it with people every time” [GP3]*

*“I think sometimes doctors will not raise it because they don’t want to annoy the patient” [GP7].*

This factor is, of course, related to the knowledge gaps and confidence issues described under the psychological capability component.

## Discussion

Using a theoretical model of behaviour, this study identified eleven key drivers of GP behaviour that impact implementation of recommended practice for diagnosis and non-drug, conservative management of knee OA in the Australian primary care setting.

Our findings suggest whilst GPs mostly know that knee OA can be diagnosed without imaging and that exercise and weight loss are recommended, their described behaviours in practice were often discordant with their own knowledge. We identified several barriers that lead to this discordance. Despite participating GPs describing OA diagnosis and management as straightforward, comments indicated incomplete or inaccurate *knowledge* of OA disease processes and prognosis, and low *confidence* with diagnosing knee OA without x-ray. Knowledge that goes beyond the general guideline recommendation to the what and why of exercise and weight loss interventions, and confidence in their ability to facilitate these interventions effectively, also appeared insufficient. Most, but not all, GPs were aware of their lack of knowledge and confidence. Feelings of being ill prepared to manage OA are consistent with other studies [7, 18, 22, 30, 31]. GPs in our study tended to adopt a simplistic model of OA which neglects the involvement of joint structures other than cartilage, promotes a biomedical model of the disease and its consequences, and may contribute to a fatalistic attitude among patients. Understanding knee OA as a problem of chronic pain involving broader psychosocial factors and as a condition that affects multiple joint structures [32] and therefore not requiring imaging for diagnosis [29], is likely to require a substantial shift in long-held beliefs and habits for many GPs.

GPs’ reflections on conversations with their patients about OA identified a reliance on terms that normalise OA (e.g. OA is to be expected with aging), and a preference for vague, general terminology such as ‘wear and tear’ to communicate the diagnosis to patients. Previous research suggests a dissonance between GPs’ rationale for avoiding articulating the diagnosis and how a vague diagnosis is perceived by patients. Clinicians play down an OA diagnosis in an attempt to facilitate acceptance and avoid upsetting patients [30]. However, using dismissive or reassuring terms over factual explanations and empathy can be interpreted by patients as the GP trivialising their problem [18, 30, 33] and lacking interest in their debilitating symptoms [30, 34]. GPs may therefore unintentionally be acting as a barrier to patients making beneficial lifestyle changes, communicating in a more top-down rather than collaborative way with patients about OA diagnosis and treatment options.

Time pressure was identified in our findings and has been widely cited previously as a barrier to GPs ability to implement CPG recommendations including facilitating lifestyle changes [22, 30, 31, 33–36]. The problem is exacerbated when patients with OA have multiple comorbidities and GP consultation length averages only 14–15 min [37]. A UK study found limited time (13 min) and the presence of multiple co-morbidities (3 conditions on average) led to GPs spending minimal time on OA management and prioritising other conditions they perceived as greater threats to patient health [33]. However, OA imposes a substantial burden on individuals and impacts health-related quality of life to at least the same degree as other common chronic diseases [38, 39]. GPs placing less importance on OA management than the patients themselves may lead to under-management in primary care and patients feeling unsupported and dissatisfied [40].

While some of the barriers to evidence-based management found in this study may be specific to managing OA, many of the barriers have also been found in previous qualitative and quantitative (survey) studies on other chronic conditions including, for example, low back pain [41–43], diabetes [44, 45], chronic kidney disease [46, 47], chronic pain [48], depression [49] and obesity [50, 51]. Frequently occurring barriers include incongruency between patient wishes and guideline recommendations [41–43, 48, 49], suboptimal practitioner skills for patient education [42, 45–47, 50] – in particular, the difficulty communicating a non-biomedical explanation of a biopsychosocial problem [41, 43, 48], difficulty ‘selling’ lifestyle change and providing support for better self-management [42, 45–48, 50, 51], frustration with patients [45, 47, 50, 51], lack of time [41, 43, 45–49, 51], limited access to other services to help with management [41, 42, 46–49], and resistance to changing practice habits [44, 49]. Collectively, these problems may reflect the challenges in managing chronic conditions within a system designed primarily to manage acute illnesses [52, 53]. On the plus side, the commonality across many conditions, especially around lacking skills and confidence in having conversations about obesity and supporting patients to increase physical activity, means that addressing some of the barriers to optimal OA care will be transferable to improving management of many other conditions and vice versa, particularly given many patients have multi-morbidity.

Our findings identified multiple interacting barriers influence GPs’ implementation of OA guideline recommendations, suggesting complex, multimodal solutions may be required. Targeted GP education and training interventions to build motivation and confidence were potential facilitators to clinical guideline adherence identified by the GPs in ours and other studies [54, 55]. Changes to the guidelines themselves may be beneficial. Currently, OA guidelines lack specific exercise and weight management recommendations and are open to variable interpretation [56, 57] potentially resulting in GPs feeling ill equipped to deliver lifestyle interventions [22]. Further research to identify optimal exercise types and dosage, and effective weight loss interventions is required, however it is currently feasible to suggest specific exercise programs based on existing exercise science and general exercise and physical activity recommendations [58, 59], and to provide guidance on how to have effective conversations with patients to facilitate adoption of lifestyle change recommendations based on principles of patient-centred care and health behaviour change [29, 60]. Guidelines could also provide clear, plain-language statements that can be readily used by GPs during consultations to help with OA management discussions [61]. This type of communication guidance has been demonstrated to facilitate GP uptake of CPG recommendations [34]. Finally, incentivisation and/or coercion-based interventions may help address motivational barriers. This is based on the work of Michie et al. who developed the Behaviour Change Wheel [16], which is an evidence-based framework for planning behaviour change interventions [24]. The framework provides a systematic and theoretically guided method for identifying the types of interventions that could be expected to be effective. Incentivisation and coercion are behaviour change intervention ‘functions’ that are suggested for addressing some of the types of barriers we found among our GP sample, namely reflective and automatic motivation barriers [16]. Incentivisation has previously been used to drive GP adoption of patient-centred care; however, results have been varied [62]. Costly pay-for-performance interventions appear to influence short-term GP behaviour change, but not long-term, and do not appear to translate to improved patient outcomes [63, 64]. Coercion in this context means creating an expectation of cost. Costs could include financial loss or negative feelings about the undesired behaviour(s). Thus, examples of coercion in our context could include reducing rebates for care consistent with the undesired behaviour, providing education about the negative consequences to patients when sub-optimal care is provided, or portraying those behaviours as ‘old fashioned’. Michie et al. [16] note that whether these functions are effective or not depends on the behaviour and the circumstances and should be thought of as options for consideration. Coercive interventions seem not to have been investigated as yet, and it may be that they are not perceived to be acceptable in this context. Utilising social influences via communities of practice or local opinion leaders, or diffusion of innovations aimed at shifting practice behaviours may be better options for addressing reflective and automatic motivation barriers [65].

Several strengths and limitations should be considered when interpreting the findings beyond the context of this study. The attitudes and beliefs of GPs willing to participate in research may not be representative of all GPs. Most GPs interviewed had at least 20 years experience and the views and practices may differ amongst GPs with less experience and/or those who completed training more recently. These, and other location-related contextual factors, including system-related findings pertaining specifically to the Australian healthcare system, should be considered when transferring findings to other contexts. However, it is likely that many findings will be relevant across contexts. In addition, the data represent only the version of GP’s perceived reality they wanted to share with the interviewer, and interpretation is influenced by the analysis team. Thus, there may be important factors that influence GP practice behaviour not detected by the study.

## Conclusion

In summary, our analysis of Australian GPs’ discussion of implementing core underutilised CPG recommendations for knee OA management identified multiple influences that impact practice behaviour. Key negative influences identified were knowledge gaps, low confidence and skill deficiencies, time and other system constraints, and the GPs’ perceived role, assumptions about patients and established habits. Positive influences include the benefits of healthy lifestyle changes for all patients, GP optimism and using a patient-centred approach. The complexity of these influences suggests complex, multi-model solutions may be necessary including changes to clinical guidelines, targeted education and training, the implementation of new models of service delivery and exploitation of positive social influences. Such interventions may help bridge the evidence-to-practice gap, which is almost certainly needed if the individual and societal burden of knee OA is to be reduced.

## Abbreviations

COM-B: Capability/opportunity/motivation - behaviour
COREQ: Consolidated criteria for reporting qualitative studies
GP: General practitioner
OA: Osteoarthritis
